# Economic estimates of invasive wild ungulate damage to livestock producers in Hawai'i

**DOI:** 10.1002/ps.8446

**Published:** 2024-10-03

**Authors:** Stephanie A Shwiff, Carolyn LW Auweloa, Kyle Caires, Greg Friel, Lauren Katayama, Zachary Munoz, Melissa R Price, Derek Risch, Mostafa Shartaj, Karen Steensma, Mark Thorne, Ray Zifko

**Affiliations:** ^1^ USDA APHIS Wildlife Services National Wildlife Research Center Fort Collins CO USA; ^2^ USDA NRCS Pacific Islands Area Range Management Honolulu HI USA; ^3^ College of Tropical Agriculture & Human Resources, Department of Human Nutrition, Food & Animal Sciences University of Hawai'i at Mānoa Honolulu HI USA; ^4^ Haleakala Ranch Maui County HI USA; ^5^ College of Tropical Agriculture & Human Resources, Department of Natural Resources & Environmental Management University of Hawai'i at Mānoa Honolulu HI USA; ^6^ Department of Economics Colorado State University Fort Collins CO USA; ^7^ Department of Geography & Environment Trinity Western University Langley Canada

**Keywords:** wild ungulate, economic, damage, invasive, livestock

## Abstract

**BACKGROUND:**

Invasive ungulates (hoofed mammals), including deer, feral pigs, feral goats, and feral sheep, are known to cause damage to agriculture, property, natural resources, and many other commodities. Most of the information regarding the economic impacts of wild ungulates is from North America, where some of these species are native. To evaluate invasive ungulate damage to livestock producers in the Hawaiian Islands, which have no native ungulates, a survey was distributed to livestock producers across the state.

**RESULTS:**

Survey results described how total annual costs are distributed among damage, control, and repairs for survey respondents, who represented a significant percentage of total ranchland acreage across the islands. The estimates, excluding fixed fence installation, revealed an annual cost to livestock producers who responded to the survey of US$1.42 million, which ranged from $3.6 million to $7.5 million when extrapolated to the entire state. The large cost contributors included damage to property, pastureland repair, control costs (excluding fencing), supplemental feed, and predation of calves by wild pigs. Additionally, producers reported spending more than $2 million in upfront fence installation costs. Most of these costs were reported by respondents on the islands of Hawai'i and Moloka'i.

**CONCLUSION:**

Study results revealed substantial damage to state livestock producers due to wild ungulates and are useful in determining an invasive ungulate management strategy that can appropriately aid the most impacted sectors of Hawai'i. © 2024 The Author(s). *Pest Management Science* published by John Wiley & Sons Ltd on behalf of Society of Chemical Industry.

## Graphical abstract

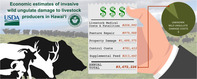

## 1 INTRODUCTION

**Figure 1:**
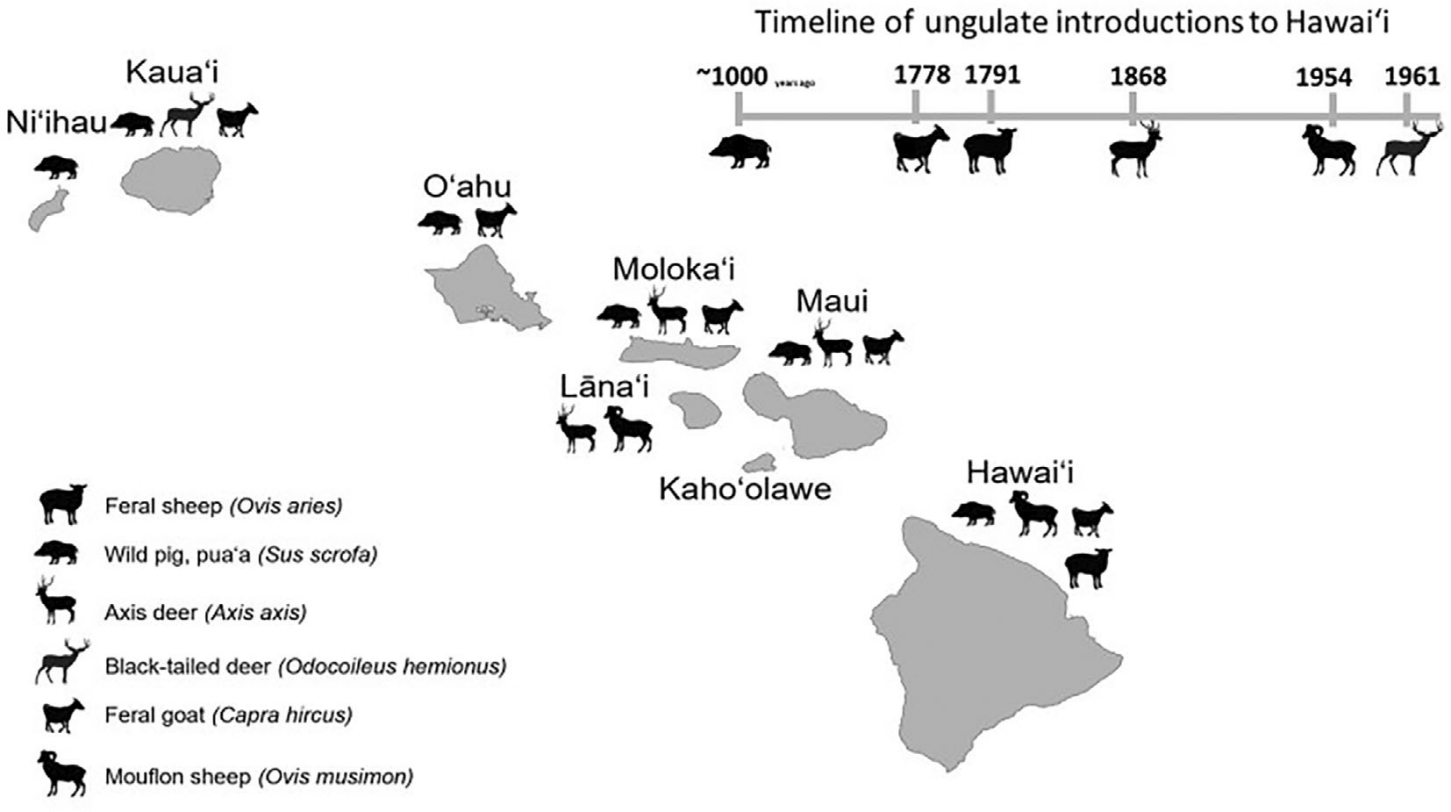
Wild ungulate presence in the Hawaiian Islands. Species include those recognized by the State of Hawai'i as game mammals. Historical introduction information obtained from Tomich (1986).

Damages from wild and invasive ungulates (hoofed mammals) to US agriculture have a cumulative annual impact in the hundreds of millions to billions of dollars.^1^, ^2^, ^3^ Invasive ungulates naturalized in the Hawaiian Islands currently include wild pigs (also known as feral pigs, wild boar, feral swine) (*Sus scrofa*), Mouflon sheep (*Ovis musimon*), feral sheep (*Ovis aries*), axis deer (*Axis axis*), feral goats (*Capra hircus)*, feral cattle *(Bos taurus)*, and black‐tailed deer/mule deer (*Odocoileus hemionus*), but not all of these occur on every island, and impacts vary by species and land system (Fig. 1).^6^, ^13^ The Hawaiian Islands lack native ungulates, but hoofed mammals have been introduced by people, starting with the introduction of domesticated pigs by Polynesian voyagers ~1000 years ago and the introduction of goats, sheep, cattle, horses, and donkeys by European and American colonizers over the past 230 years.^4^ Human manipulation of the Hawaiian landscape in the last few centuries, combined with the novel disturbance regime brought by hoofed mammals, have significantly altered native Hawaiian ecology.^5^

By the 1930s, just about 291,000 acres in the Hawaiian Islands were under intense industrial agricultural production, most of which was sugar and pineapple, and another 2 million acres were grazed by cattle.^6^ As sugar and pineapple production declined and eventually ceased, large areas of previously intensively cultivated and managed land have been left fallow and unmanaged.^7^ Public access to these lands for hunting purposes has become more limited in the shift from plantation management to new land ownership, reducing hunting access to this land.^8^ Reducing hunting on managed lands may expand the undisturbed habitat for introduced ungulates and has likely supported increased populations of wild invasive ungulates, further impacting native ecosystems and agricultural production.^4^ State agency authority over game species in Hawai'i, in contrast to the continental United States, does not extend to private lands (Hawai'i Revised Statutes §183D), creating a significant management burden for producers on private lands. While numerous studies have explored the impacts of invasive ungulates on native ecosystems in the Hawaiian Islands,^5^, ^9^, ^10^, ^11^, ^12^ few if any studies to date have assessed damage to agricultural production.^13^ Thus, economic estimates of wild ungulate damage to agricultural production in the Hawaiian Islands are largely unknown, alongside the heterogeneity in damage, implications for biosecurity and food security, and control costs across species, land types, and islands.^14^, ^15^, ^16^, ^17^ Not only is this information critical to devising policy and management solutions for wild ungulate populations, but given the isolation of the Hawaiian Islands, there is a critical need to improve biosecurity to ensure food security by increasing the efficiency of local food production.^18^, ^19^

After the introduction of Polynesian pigs, subsequent introductions of goats, sheep, cattle, and deer were often allowed to roam free and were protected by a kapu, or taboo, strict code of conduct that prohibited their harm in order for their populations to establish.^6^ With the introduction of invasive earthworms and dung beetles by Europeans, the environment of the Hawaiian Islands changed, providing pigs with high‐protein sustenance, leading to significant population increase and subsequent ecological damage, such as spread of invasive species,^20^, ^21^ predation of ground‐nesting birds and native plants, increased erosion through digging behaviors, and changes in nutrient cycling.^22^ Today feral pigs are well‐recognized globally as invasive ecosystem engineers (IEE) that have led to extinctions and population declines in native species across the Pacific Islands, as well as globally.^23^ IEE are a type of invasive species that can have a significant impact on the environment, either physically or chemically. These species can alter the physical–chemical structure of ecosystems, which can change the conditions for many species that live there.

Damage by other introduced wild ungulates to Pacific Island ecosystems is also well documented. Introduced in 1957, mouflon have become a problematic invasive species in the Hawaiian Islands of Lanai and Hawai'i.^13^ They have degraded native ecosystems through browsing, bark stripping, and trampling, which threatens the fragile native flora. Domestic goats, initially brought to the Hawaiian Islands by Captain Cook in 1778,^24^ have become wild and feral throughout many islands, and have earned the label of ‘the single most destructive herbivore’ in island ecosystems globally.^25^ Axis and black‐tailed deer cause damage through browsing and habitat degradation but differ in distribution, with axis deer preferring drier areas and black‐tailed deer occupying wet forests (Risch *et al*., under review). When axis deer populations exceed forage availability they will range well beyond their preferred habitats, into areas of high elevations and high rainfall. In extreme conditions, axis deer consume all palatable vegetation, strip bark from trees, and girdle and kill mature trees.^26^ Concentrated in fragile, wet areas below cloud forests, deer create trails through thick vegetation, causing soil compaction, reducing ground mosses, and increasing runoff and erosion.^26^ Combinations of these ungulates in some places has led to regime shifts from native‐dominated to invasive‐dominated ecosystems, as well as the degradation of coral reefs and fisheries, alongside damage to archaeological sites.^26^, ^27^

While the impacts of wild ungulates on native ecosystems in the Hawaiian Islands have been well documented, impacts on agricultural production have received less attention. Direct impacts include disease transmission, predation of young, and hybridization.^13^ Wild ungulates, especially wild pigs, are capable of vectoring numerous diseases to livestock (e.g., African swine fever, classical swine fever, and foot‐and‐mouth disease).^28^ Pigs are a highly gregarious and social species, and interact with livestock,^29^ companion animals,^30^, ^31^ and humans.^32^ Indirect impacts on agricultural production may also be substantial due to a decrease in water and forage availability, change in nutrient cycling, erosion, and the spread of noxious and invasive weed species.^2^ Historical references to goats being rounded up and destroyed or harvested cite competition with livestock for forage as being the main motivation.^33^ Anderson *et al*.^26^ also cite deer encounters in cane fields and competition with cattle for pasture on rangelands.

While farmers across all the islands have long complained of the damage pigs cause to their crops and operations, there are no comprehensive economic estimates of wild ungulate damage to livestock producers in the Hawaiian Islands (G. Friel, 2023, pers. obs.). Moreover, existing estimates from North America may not extrapolate to the Hawaiian Islands due to differences in geography, environment, social norms related to ungulates, regulations, and a history of invasive ungulates. However, studies suggest multiple types of costs may be incurred from the presence of wild ungulates. Control costs of wild pigs in national parks in the Hawaiian Islands are approximately $450,000 annually (Roldolfo Zuniga, unpublished data).^34^, ^35^ Other research focusing on wild pig damage in the Hawaiian Islands reveals that 80% of soils inhabited by wild pigs are disturbed^36^ and therefore susceptible to invasive plants and soil erosion,^35^ incurring further costs for agricultural producers.

This manuscript summarizes a recent survey‐based effort to fill this gap. We proceed with a discussion of the survey methodology, survey instrument, and survey distribution. Results are then presented with a focus on three key objectives: (i) the types of wild ungulates causing damage to livestock producers, (ii) the severity of damage that livestock producers experienced, and (iii) the types and perceived effectiveness of the methods used to mitigate wild ungulate damage. Presentation of the results is followed by a discussion of their implications. Ultimately, the information we present may highlight the impact of wild ungulates on Hawaiian livestock producers, and enhance private and public control efforts, allowing resources to be allocated to the most severe problems.

## 2 METHODS

A survey was designed to allow estimation of the value of wild ungulate damage and control for livestock producers in the Hawaiian Islands, including the severity of damage and damage costs, control costs, and descriptive data. The University of Hawai'i‐Manoa Institutional Review Board (IRB) reviewed all survey materials and approved the survey on 26 September 2022 (IRB #2021‐00877). Questions were based on previously deployed surveys in multiple geographic regions, including Anderson *et al*.^2^ and McKee *et al*.,^3^ and were adapted to be appropriate to the context of the Hawaiian Islands and the goals of the present study.

Data were collected through a self‐administered questionnaire hosted on Qualtrics (Qualtrics XM, Provo, UT, USA), an online survey platform, by the University of Hawai'i at Mānoa. A judgment sampling method was employed.^37^ Judgment sampling is a non‐random sampling method in which the sample is selected based on the researcher's knowledge of the units to be sampled. For this sample, the elements of the population selected for inclusion were known livestock producers on the islands. This method is used when a standard sampling method cannot be applied or is not appropriate given the aims of the study. As there is currently no known email (listserv), telephone, or mail distribution list for the livestock producers on the islands, we identified representative subsets of the larger population through groups such as the Hawaiian Cattlemen's Council listserv (email distribution list), the Hawai'i Range and Livestock Management News newsletter listserv, and the Hawai'i Farm Bureau listserv.

The survey instrument was designed to elicit a range of values associated with wild ungulate presence. Although tailored in part to specifics of livestock production, the survey also collected information on property damage, control costs, and hunting practices. In addition to questions regarding potential disease spread from wild ungulates and related concerns, as well as pasture damage, producers were asked about livestock loss from depredation, disease, and other causes on their facility. Additionally, they were asked to report the costs of medical treatments and veterinary services related to wild ungulate contact with their livestock. Respondents first indicated their geographic location, which resulted in the electronic survey subsequently restricting which wild or feral ungulate species were included as potential responses to minimize inadvertently inaccurate responses as the presence of species varies among islands.

**Figure 2:**
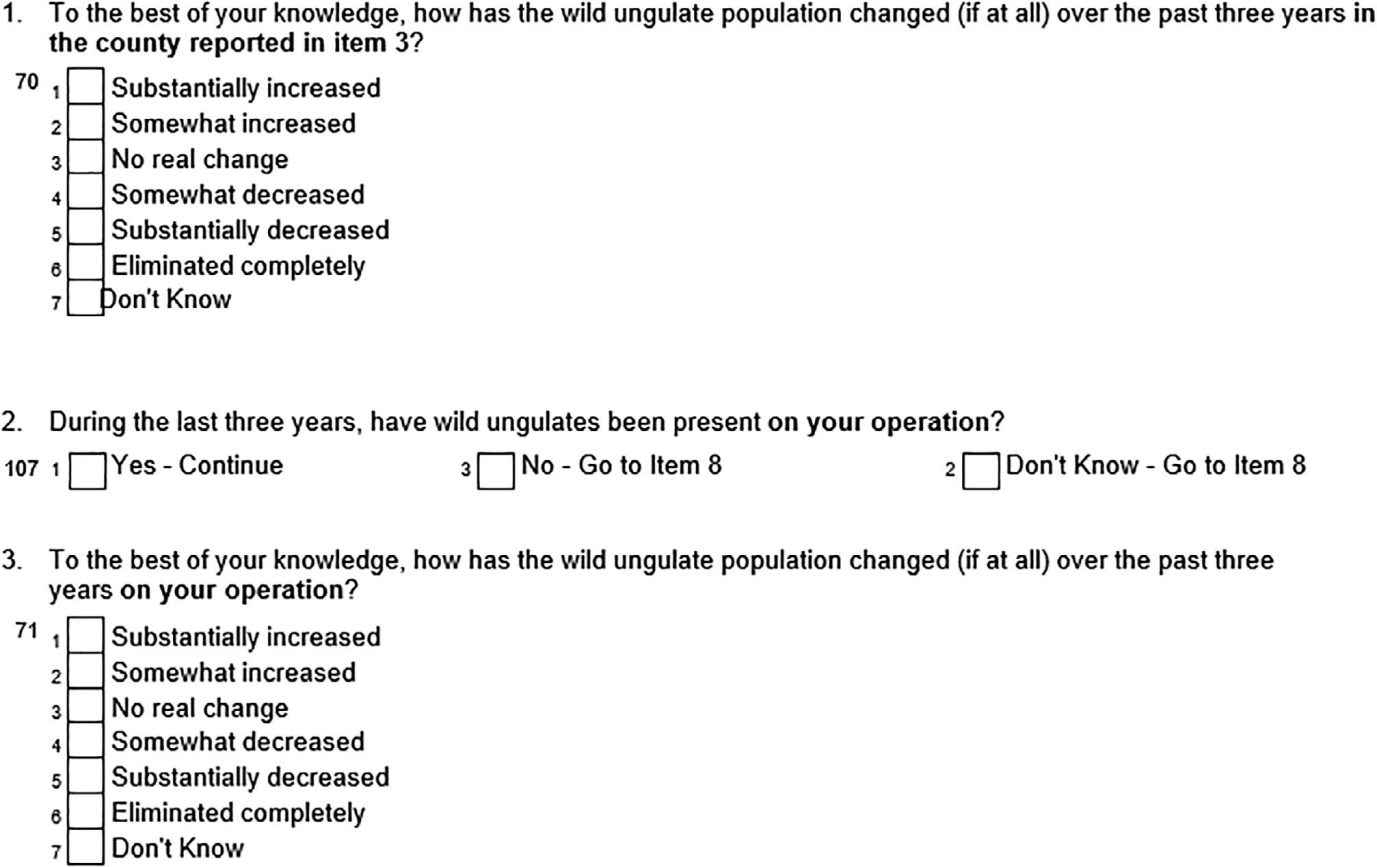
Survey questions related to wild ungulate presence in the county and on the operation.

There were three core types of information collected by the survey: information on wild ungulate presence, damage, and control methods. The first type of information was the presence and recent changes in the population of wild ungulates in the producer's county and on their operation (Fig. 2). Wild ungulate presence provides a general indication of the economic threat they pose in the area through either direct damage or the risk of disease transmission.

**Figure 3:**
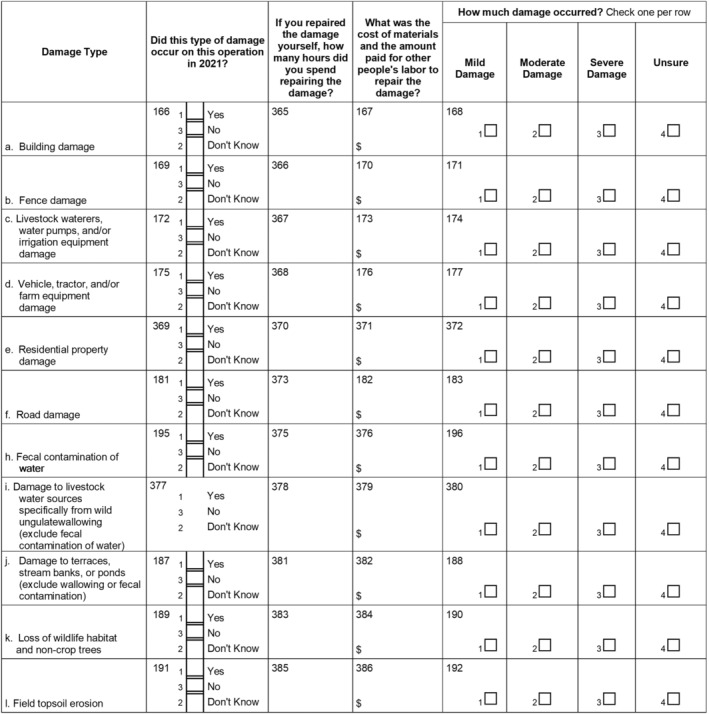
Questions related to wild ungulate damage to operational resources.

The second core type of information collected was on wild ungulate damage to property (e.g., buildings, vehicles, equipment, roads, fecal contamination of resources), livestock losses, disease implications, veterinary care, medical costs, and pasture related damage. Property damage costs were assessed by asking producers to report damage due to destruction of structures, impacts on water resources used by livestock, and damage to pastures, trees, habitat, and other damage (Fig. 3).

**Figure 4:**
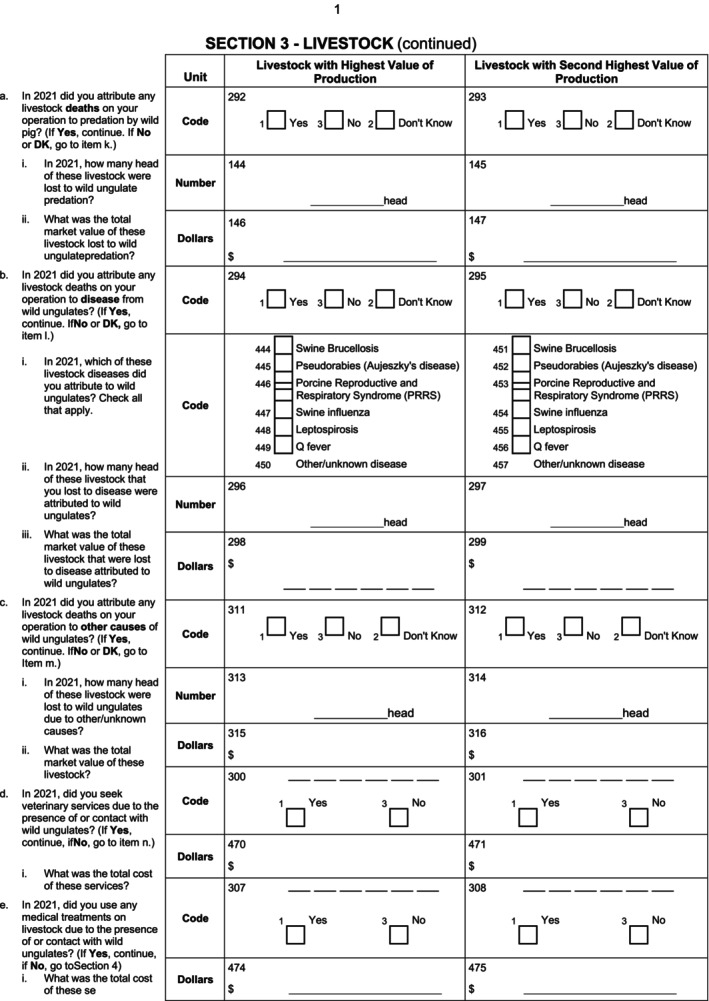
Questions related to livestock losses, disease, and veterinary and medical costs.

Sections on livestock losses, disease transmission risk, and veterinary and medical costs followed (Fig. 4). Livestock medical and veterinary care was calculated as a sum of predation by wild pigs, disease, and medical and veterinary costs, which were all measured from value lost/costs associated with their highest and second‐highest value livestock. As such, these do not capture the total costs of all their livestock, only their livestock with the highest value of production.

**Figure 5:**
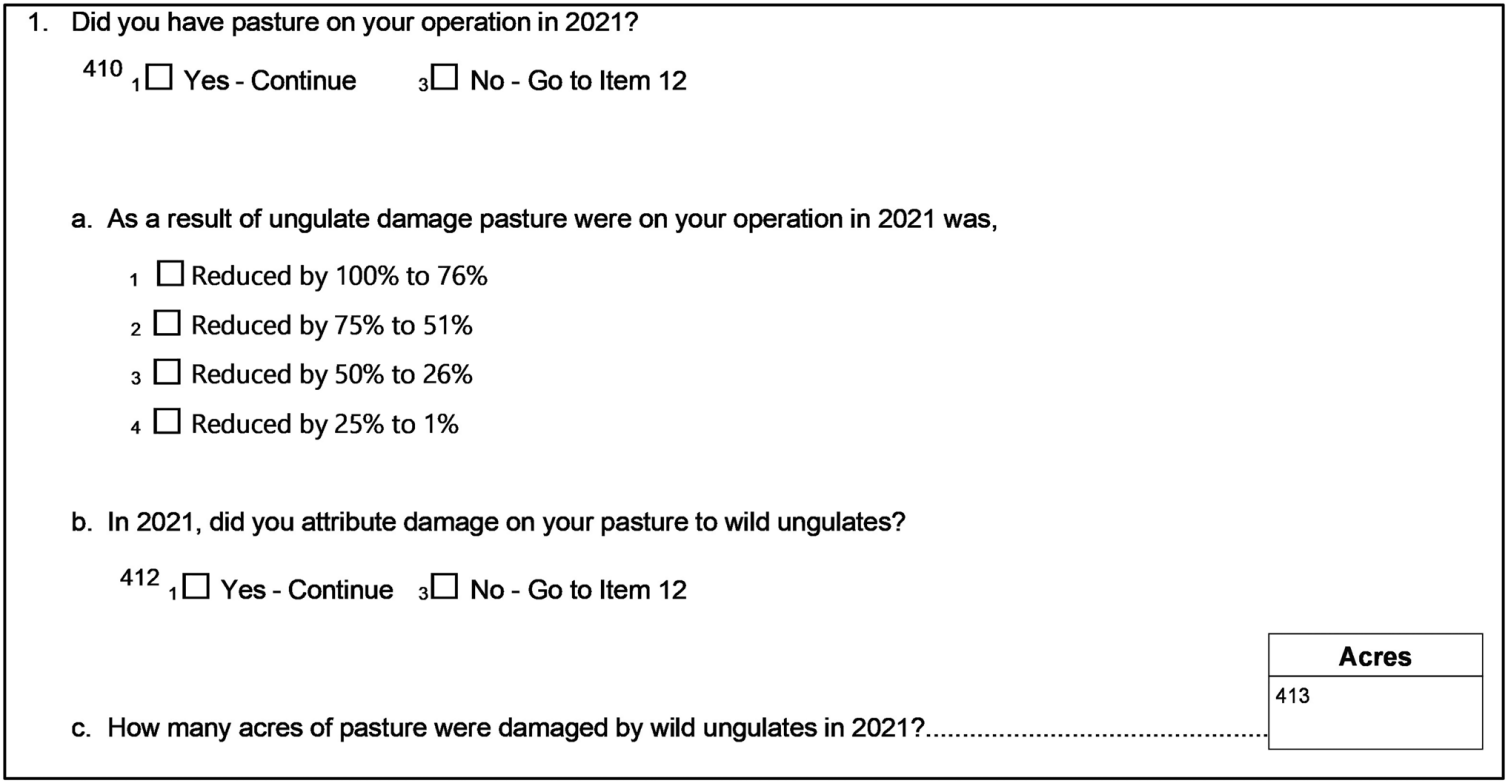
Questions related to wild ungulate damage to pasture.

Pasture damage was assessed with relative pasture losses (Fig. 5). The economic value of forage lost to wild ungulate grazing was not estimated.

**Figure 6:**
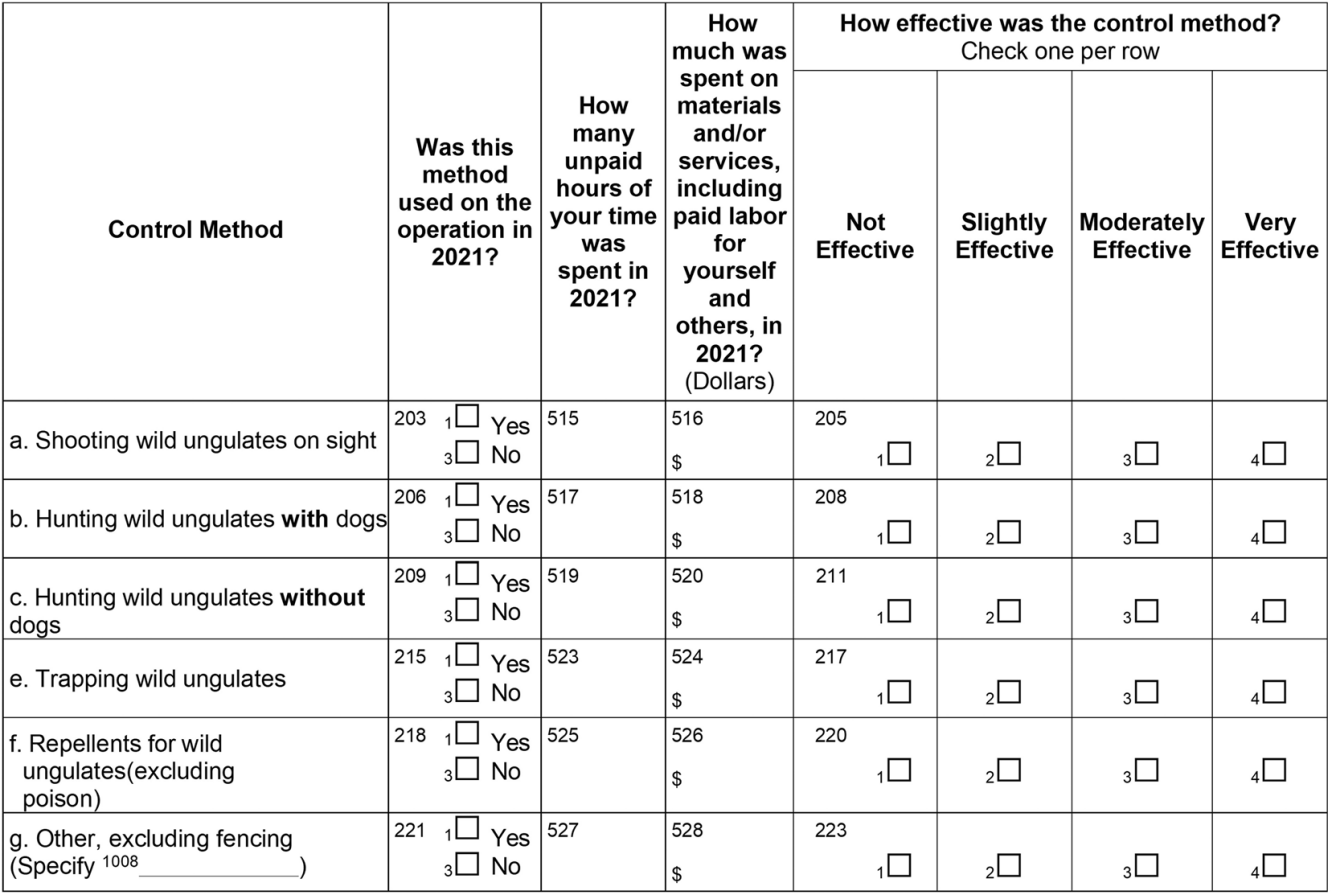
Questions related to wild ungulate control methods and associated costs.

Control costs were calculated by summing all forms of control methods utilized by producers, including annual fence installation costs (Fig. 6). The annual fence cost was the total cost to install a non‐electric fence divided by the estimated years of use, provided by the producers. For the survey acres covered (39%), we determined a weighted average of $4.81/acre in damage plus control costs. The total amount of pasture acreage across all the islands is approximately 765,579 (Hawai'i Department of Agriculture, https://hdoa.hawaii.gov/salub/).

We asked producers to report information from 2021 because that was the most recent complete production year at the time that the survey was administered and was arguably easy for producers to self‐report. Despite the potential inaccuracies associated with relying on self‐reported damages, we chose this design for several reasons. First, self‐reporting of wildlife damage to agriculture is common and has been shown to be accurate way to reflect actual damage suffered by producers.^38^, ^39^, ^40^, ^41^ Second, livestock values can vary substantially according to the region and type of livestock (even with specific categories). We believed it preferable to rely on Hawaiian producers who have knowledge of local values and prices rather than making inferences from broader pricing statistics. Finally, even if producer perceptions vary from reality, producers often make decisions based on their perception of reality.

## 3 RESULTS

**Figure 7:**
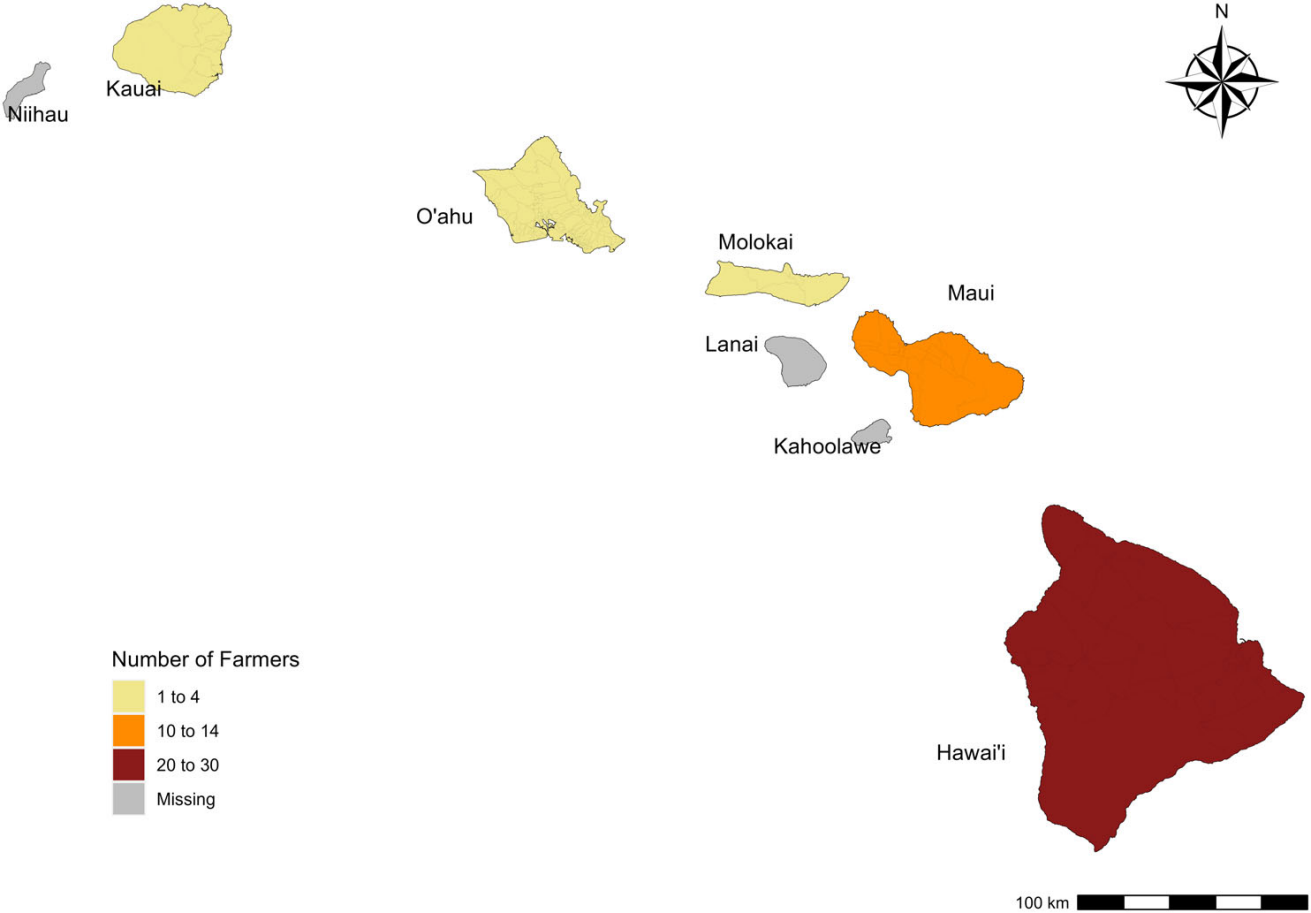
Distribution of survey respondents.

The survey was administered from February 2023 to June 2023. It received 113 responses, 68 of which were complete and met validation requirements. These validation requirements included surveys that could not be used consisted of not applicable (NA) as a response to all questions, respondents who did not reside in Hawai'i, or respondents provided the same value for every response. Given these considerations, out of the 68 responses 45 were usable from the targeted audience of livestock producers (Fig. 7).

These 45 surveys were received from five islands, representing approximately 39% of all reported pasturelands in the Hawaiian Islands (Table 1). Surveyed acres covered a significant amount of the pastureland for the islands of Hawai'i (43%) and Maui (46%), but lower percentages for O'ahu (18%), Kaua'i (6%), and Moloka'i (6%). The survey results were compared to total pastureland data as reported by the Hawai'i Department of Agriculture in the latest reporting year (Hawai'i Department of Agriculture, https://hdoa.hawaii.gov/salub/). There were no participants from Ni'ihau or Lāna'i in our survey. Regarding pasture estimates, we assumed for this analysis that all of the land reported in the survey was designated as pastureland.

**Table 1:** Surveyed pastureland acres *vs* total pastureland acres by island

Island	Surveyed acres	Total acres	Surveyed acres as % of total
Hawai′i	235,569	552,091	43%
Kaua′i	2500	42,345	6%
Maui	53,316	115,241	46%
Moloka′i	2410	37,867	6%
O′ahu	3183	18,035	18%
Total	296,978	765,579	39%

Most of the respondents reported that wild ungulate presence in their county and on their operation had increased over the last 3 years. On Hawai'I, 96% of respondents indicated that wild ungulate presence had either somewhat increased or substantially increased in their county and 89% reported an increase on their operation. For Moloka'i and O'ahu, 100% of respondents reported that wild ungulate presence had increased in their county and on their operation. For Maui, 100% indicated an increase in their county and 89% on their operation.

For most of the respondents, the livestock of concern was beef cattle. The average number of livestock reported by respondents was 877 (range 0–15,125, standard deviation = 2442). Beef cattle, goats, and sheep were the top three livestock species raised by respondents, and on average respondents owned two livestock species. On Hawai'i most producers raised cattle, with goats ranking second in terms of overall number of animals on their facility. On Kaua'i and Moloka'i surveyed producers primarily raised cattle. On Maui, producers reported that cattle were the primary livestock produced, with sheep ranking second which was the exact opposite to O'ahu.

Wild pigs (present on all islands except Lāna'i) and axis deer (present only on Maui and Moloka'i) were reported to cause the most damage to property and operations (Table 2). Additionally, among livestock producers on the island of Hawai'i (*n* = 28), significant damage was reported from mouflon sheep (17% of respondents), feral sheep (14% of respondents), and feral goats (21% of respondents). Producers from Maui reported a high degree of damage by axis deer (70% of respondents) and feral goats (20% of respondents).

**Table 2:** Percentage of farmers reporting severe damage by wild ungulate

Island	Feral pigs	Axis deer	Mouflon sheep	Feral sheep	Feral goat	*n*
Hawai'i	76%	NA	17%	14%	21%	28
Kaua'i	0%	NA	NA	NA	0%	1
Maui	40%	70%	NA	NA	20%	10
Moloka'i	75%	100%	NA	NA	0%	4
O'ahu	100%	NA	NA	NA	0%	2

NA = species does not occur on this island.

**Figure 8:**
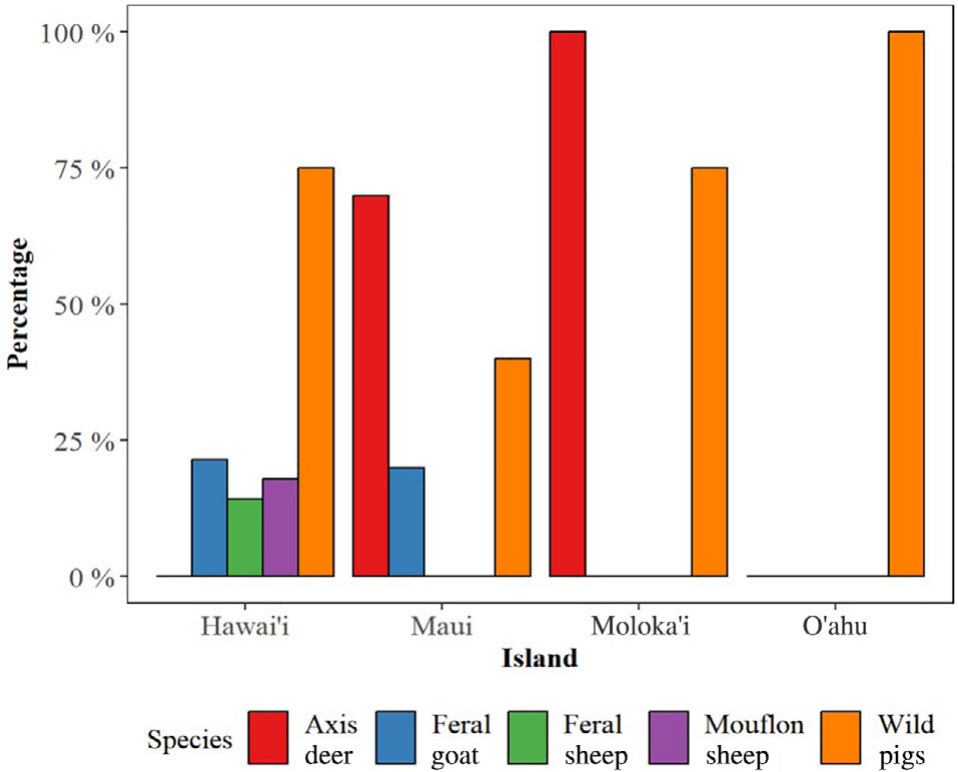
Percentage of moderate to high levels of damage by wild or feral ungulate species.

On a scale of 1 (causing minimal damage) to 5 (causing the most damage) based on the damage to their operations, most respondents rated feral pigs and axis deer as causing damage at a scale of 4 or 5 (Fig. 8), while feral sheep, goats, and mouflon sheep were rated lower. Of the islands from which responses were received, axis deer are only present on Maui and Moloka'i, and feral sheep, mouflon sheep, and hybridized feral and mouflon sheep are only present on Hawai'i island.

The total annual cost of wild ungulates summed across all ranch operations represented in the survey was reported to be $1.42 million, with most of the costs resulting from direct damage and control costs (Table 3).

**Table 3:** Total annual costs of wild ungulates

Island	Property damage	Pasture repair	Control	Supplemental feed	Livestock medical/predation	Total	Surveyed Acres	Total pastureland (acres)	Surveyed acres (% of total acres)	Damage cost per surveyed acres
Hawai′i	$394,865	$138,650	$130,360	$17,500	$46,070	$727,445	235,569	552,091	43%	$3.09
Kaua′i	$1000	0	0	0	0	$1000	2500	42,345	6%	$0.40
Maui	$79,000	$66,000	$154,860	$38,804	$7500	$346,864	53,316	115,241	46%	$6.51
Moloka′i	$101,320	$175,400	$2500	$26,500	$2500	$308,220	2410	37,867	6%	$127.89
O′ahu	$475	$100	$15,400	0	$25,000	$40,975	3183	18,035	18%	$12.87
Total	$577,360	$380,150	$303,120	$82,804	$81,070	$1,424,504	296,978	765,579	39%	$4.80

Additionally, livestock producers spent around $2.03 million in fence installation costs. The largest costs were from property damage, control, and pasture repair (Table 3), but respondents were only asked to consider the two highest value livestock species, which were less than all livestock species raised for nearly all the producers. The summation of damage costs by island provides the opportunity to determine damage costs per acre by island. Combining the damage costs per acre with total acres allows for the extrapolation of these costs for the entire state. Two extrapolations were made. First, we utilized the damage cost per acre for the total number of surveyed acres ($4.80) and multiplied this by the total number of pastureland acres in Hawai'i (756,579) to determine an annual estimate of over $3.6 million in damage and control costs to Hawaiian livestock producers annually. Second, we utilized the calculated damage cost per acre for each specific island and then extrapolated that value across only that island and then summed across the islands. Extrapolating costs this way led to an overall annual cost to the state of over $7.5 million.

**Figure 9:**
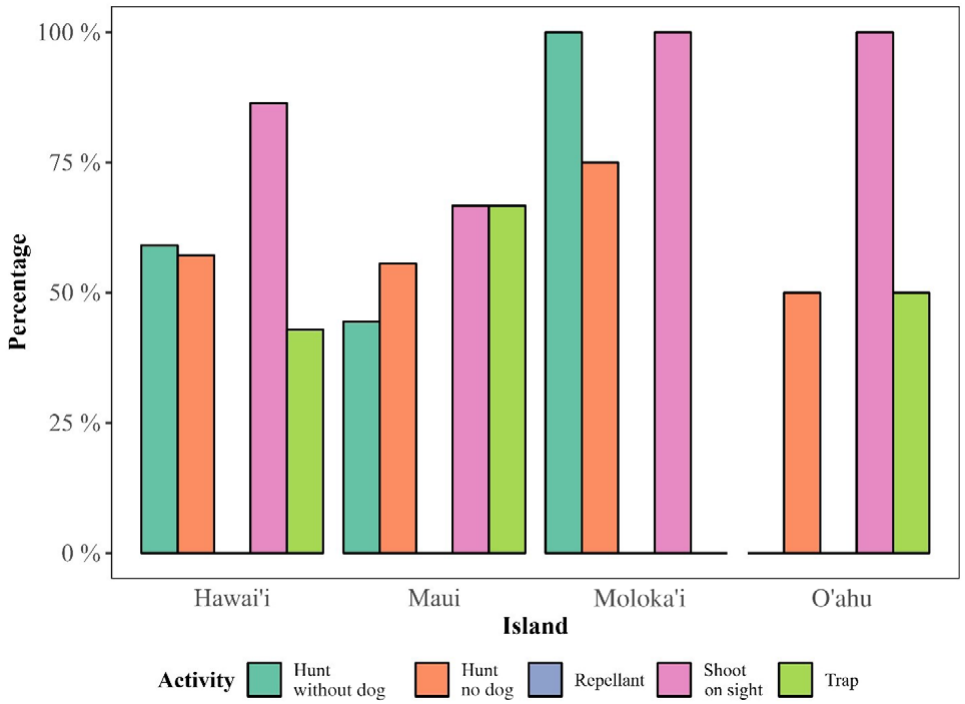
Percentage of control methods utilized by island and activity. [Correction added after first online publication on 11 October 2024; Figure 9 has been updated.]

Producers could utilize multiple methods of control so percentages will not add to 100%. The largest cost of control, other than the installation of non‐electric fences, was reported from shooting wild ungulates. Excluding fencing, a variety of control methods were used, including (listed in order of respondent preference) shooting on sight, hunting with dogs, hunting without dogs, and trapping, but no respondents used repellent. Examining this same information by island reveals that producers prefer control methods suitable for the type of invasive ungulates that occur on that island (Fig. 9).

**Figure 10:**
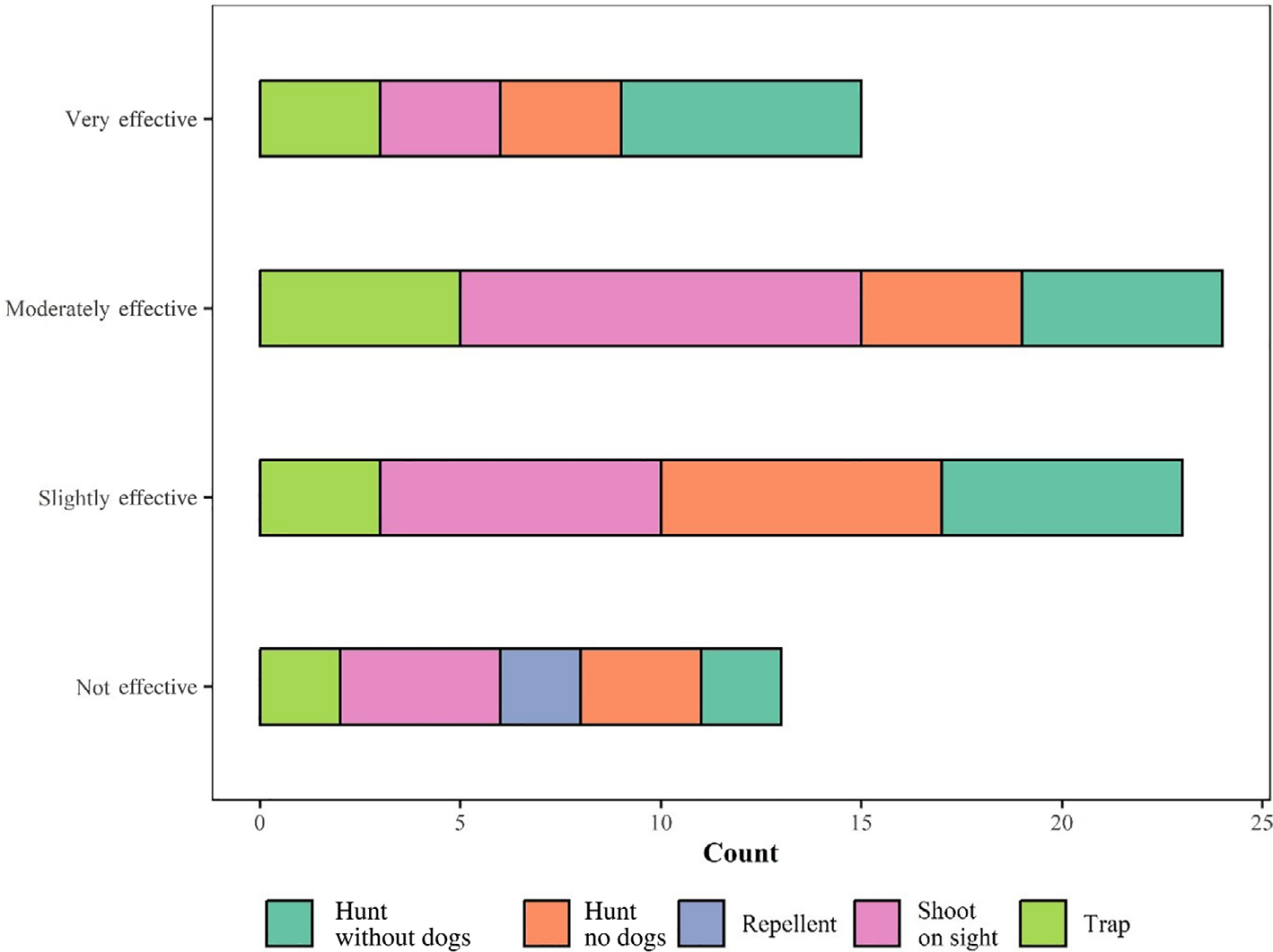
Perceived effectiveness of control methods other than fencing.

To a lesser extent producers reported that trapping was effective, and the use of repellents was viewed as not effective. Respondents reported that shooting and hunting with dogs were the most effective control methods with respect to reducing wild ungulate damage (Fig. 10).

## 4 DISCUSSION AND CONCLUSION

Invasive and feral ungulates are well known to cause substantial damage to agricultural operations globally,^23^, ^42^ but until now, no study had quantified the extent of the economic impact on livestock production in the Hawaiian Islands, which lack native hoofed mammals. We found that feral pigs and invasive axis deer cause substantial physical and economic damage to livestock production on the islands on which they occur. The total annual cost of feral and wild ungulates to livestock production across the Hawaiian Islands is estimated to range from $3.6 million to $7.5 million annually, with most of the costs coming from direct damage and control, as well as fence construction. Efforts are being made to try to rein in these damages and manage populations, including increased hunting, fencing, and commercial harvesting of ungulates where possible and feasible, but agricultural producers are still bearing the brunt of the costs created by overpopulation of these ungulates.

Damage was not equally distributed among islands, a result that is not surprising given that most ranching operations are on the island of Hawai'i, the largest island in the Hawaiian Archipelago (Hawai'i Department of Agriculture, https://hdoa.hawaii.gov/salub/), and the feral ungulate assemblages differ by island. The sum of property damage costs was the largest on the island of Hawai'i, which is consistent with the fact that most of the survey respondents were from this island and roughly 39% of the total pastureland was accounted for from the survey respondents.

Respondents on all islands reported moderate to high damage to livestock production from feral pigs, with the highest reports of damage from respondents on the island of Hawai'i. Producers tend to underestimate the costs associated with wild pig damage by a factor of three,^43^ indicating that wild ungulate damage to Hawai'i could potentially range from $10 million to over $20 million annually. Although the island wide extrapolated estimates from this study are significant and ranged from $3.6 million to $7.5 million, these annual costs likely underestimate the total cost impact to ranchers, for several reasons. First, respondents were only asked to consider their two highest valued livestock enterprises, which were less than all livestock species raised for nearly all the producers. Second, the economic burden of wild ungulate damage to pasture and property on livestock operations is not limited to lost production or increased production costs; it also includes the substantial additional cost of control efforts and risk of disease transmission and less tangible ecological costs such as the value of lost soil, reduced soil health and reduced hydrologic function.

Many producers reported applying a suite of control methods, the most common being shooting wild ungulates on sight and hunting with and without dogs. Shooting on sight was also perceived as the most effective in comparison to the other methods, similar to previous studies.^3^ Trapping and the use of repellants were reported as less commonly used and were also perceived as less effective. Producers continue to employ methods such as trapping even though these methods are perceived as less effective, in part because these methods can be used concurrently with other methods such as shooting on sight and hunting and because wild ungulates adapt to the use of any particular method over time, emphasizing the importance of not only using a suite of methods but developing new methods over time. More research on effective control methods or combinations of control methods could potentially benefit producers in their fight against wild ungulate damage. Furthermore, given that hunting pressure and the presence of hunting dogs may cause ungulates to change their distribution and behavior,^44^ and the need for increased fencing, collaboration among land managers is likely critical to successful implementation of these methods.

There were several limitations to our study. First, producers may not have accurate perceptions of damage. Such biases may be intentional or unintentional.^1^ Also, respondents may have been more likely to incur damage than non‐respondents. We assumed that total reported acres of operation were all pasture, which may overestimate the acreage devoted to pasture since some producers may be growing crops or using silvopasture techniques. This survey was also aimed at livestock producers, so crop losses are not represented here. Determining pasture losses by the value of forage lost is also difficult to gather as Hawai'i producers generally do not feed hay or lease ground on an animal unit month basis, terms and measures continental ranchers may be more apt to use to estimate loss. Costs as represented by forage loss may be estimated due to reduced herd size, lost grass‐finished opportunities, or early weaning, but inclusion of these metrics was outside the scope of this initial paper.

The economic value of ecological damage is also not estimated here, but future studies could synthesize this work with ecological impacts. Overpopulation of wild ungulates, particularly when it leads to overgrazing, causes ecological damage in the form of soil loss due to wind and water erosion, and subsequent loss of production potential, air quality impacts, water quality impacts, reduced soil health, reduced hydrologic function of working lands, reduced pasture quality due to increase in unpalatable species, and impacts on coral reefs, which leads to impacts to near‐shore fisheries and over the long term can lead to loss of shoreline protection.^45^, ^46^, ^47^, ^48^ Another social cost that is difficult to measure is the decreased food security and potential loss of water security borne by the communities of Hawai'i as the lands are degraded and there are fewer and fewer farmers and ranchers due to the increased cost of farming, which is influenced at least in part by problems caused by overpopulation of wild ungulates.

Our research to quantify wild ungulate damage to the Hawaiian Islands suggests that improved ungulate control may play a crucial role in food safety and safeguarding livestock, as well as promoting a One Health approach to management that considers the interconnections between humans, wildlife, and the environment. Targeted, well‐informed, collaborative, and consistent management of invasive wild ungulates necessitates an understanding of their economic impact on livestock producers. By understanding this damage, it is possible to provide management solutions that are not only economically viable for the producer, but are considerate of the fragile island ecosystem.

## CONFLICT OF INTEREST STATEMENT

The authors declare no conflict of interest.

## Data Availability

The data that support the findings of this study are available on request from the corresponding author. The data are not publicly available due to privacy or ethical restrictions.
